# Determination of the photon beam attenuation by the BrainLAB imaging couch: angular and field size dependence

**DOI:** 10.1120/jacmp.v10i3.2979

**Published:** 2009-06-02

**Authors:** Christopher F Njeh, Timothy W Raines, Mark W Saunders

**Affiliations:** ^1^ Radiation Oncology Department Texas Oncology Tyler TX 75702 USA

**Keywords:** carbon fiber couch, attenuation, IGRT, photon beams, IMRT

## Abstract

Highly attenuating radiation treatment couches are no longer useful in the present era of IMRT‐ and IGRT‐utilizing radiotherapy. High tensile strength and low density carbon fiber couch tops present a useful alternative. The objective of this study is to quantify the attenuation of megavoltage photons through BrainLAB imaging couch top and headrest at various angles and field sizes. At normal incidence, the couch attenuated 6 MV photons by 4.9% and 3.4% for $5\times 5\;{\text{cm}}^{2}$ and $10\times 10\;{\text{cm}}^{2}$ field sizes, respectively. The headrest, alone, attenuated 6 MV photons by 2.5% and 1.6% for the same field sizes. There was no significant attenuation of the 18 MV beam by either the couch or the headrest. We further found attenuation to be gantry angle dependent, with the highest attenuation recorded at 120° – at which angle the couch attenuated the 6 MV photon beam by 10% for the $5\times 5\;{\text{cm}}^{2}$ and 8.3% for the $10\times 10\;{\text{cm}}^{2}$ field sizes. Similarly, an 18 MV photon beam was attenuated by 3.6% and 3.4% for the $5\times 5\;{\text{cm}}^{2}$ and $10\times 10\;{\text{cm}}^{2}$ field sizes, respectively. The highest attenuation for the headrest occurred at 110° gantry angle: for the 6 MV photon beam, the headrest attenuation was 6.3% and 5.6% (for the $5\times 5\;{\text{cm}}^{2}$ and $10\times 10\;{\text{cm}}^{2}$ field sizes, respectively); for the 18 MV, attenuation was 2.3% (for the $5\times 5\;{\text{cm}}^{2}$ field size) and 2.1% (for the $10\times 10\;{\text{cm}}^{2}$ field size). It would appear, therefore, that BrainLAB imaging couch and headrest in IMRT with posterior beams results in significant decreases in the dose delivered to the target.

PACS number: 87.56.Da

## I. INTRODUCTION

Image‐guided radiation therapy (IGRT) and intensity‐modulated radiation therapy (IMRT) have revolutionized the way cancer treatment is delivered. These developments have engendered other requirements. The use of IMRT fields allows for highly conformal dose distribution to the target while reducing the dose to adjacent organs at risk.^(1)^ The design of the most optimal plan largely depends on freedom in beam incidences that can be realized by a combination of gantry and couch rotations. With these degrees of freedom, there is a possibility for the beam to pass through the couch before entering the patient, resulting in unacceptable distortion of the intended dose distribution.

The introduction of IMRT made it possible to increase the dose to the target volume. However, to limit normal tissue toxicity, treatment margins have to be decreased.^(^ ^2^ ^,^ ^3^ ^)^ In order to reduce treatment margin, the issue of organ motion needed to be addressed, so that the location of the target could be accurately determined during treatment. Target motion, for example, can cause a geometric miss in dose delivery;^(4)^ hence, accurate radiation therapy requires knowledge of the exact location of the tumor at the time of treatment. To address the problem of organ motion, many imaging techniques have recently been introduced to track such motion. Delivery of treatment using these techniques is collectively called Image‐Guided Radiation Therapy (IGRT). ^(5)^ These new imaging techniques, such as cone beam CT and orthogonal X‐ray projections, might intersect the couch when acquiring images. Hence, it is apparent that the material making up the couch is important both for online imaging and for patient dose delivery. Traditional high‐absorption couches requiring restricted gantry angles for radiotherapy^(6)^ are no longer acceptable in clinical practice. Potentially translucent carbon fiber has proven to be the material of choice.^(7)^

Carbon fibers are materials with high tensile strength and rigidity, are extremely light and have low density.^(7)^ They are usually prepared in composite form. Carbon fiber couches are usually made in the form of fat panels, each consisting of two carbon fiber plies separated by a layer of filler substance. The filler adds extra strength to the material by introducing a gap between the two sheets of carbon fiber.^(^ ^8^ ^,^ ^9^ ^)^ In addition to these properties, carbon fibers have been shown to be more radio‐translucent than conventional materials used in the construction of radiotherapy devices.^(7)^ Studies have shown that attenuation of high‐energy photon beams by carbon fibers is insignificant compared to hardboard, copolyester and polymethylmethacrylate (PMMA).^(^ ^8^ ^–^ ^10^ ^)^ Varieties of carbon fiber couches and inserts are being used in clinical practice: Sinmed Mastercouch (Sinmed BV, Reeuwijk, The Netherlands),^(11)^ Siemens IGRT carbon fiber tabletop (Siemens AG, Berlin, Germany),^(12)^ and MED‐TEC Inc. couch (Iowa, USA). Although the transmission properties of carbon fiber couches have already been reported by a few authors,^(^ ^9^ ^,^ ^11^ ^–^ ^13^ ^)^ it is still worthwhile to determine these effects for a newly‐designed table. This is because attenuation will be impacted by construction features such as thickness of the carbon fiber layers and the inserted filler substance.^(^ ^8^ ^,^ ^9^ ^)^

The objective of the current study is to quantify the attenuation of megavoltage photons when propagating through BrainLAB's imaging couch (Feldkirchen, Germany) at various angles and field sizes. These results will allow us to better model the perturbation of megavoltage photons by the couch in the treatment planning system and, as a result, more accurately determine patient dosage.

## II. MATERIALS

### A. BrainLAB's imaging couch top

**Figure 1:**
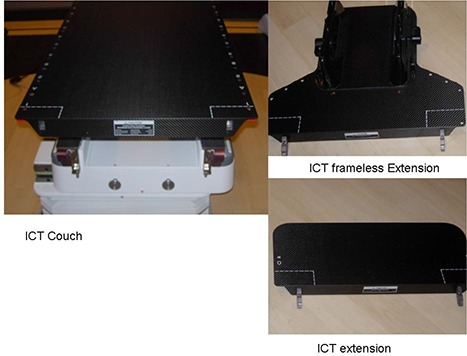
Photographs of the BrainLAB imaging couch top (ICT), extension, and frameless extension.

BrainLAB's imaging couch top (ICT) is a carbon fiber radiation therapy table (Fig. 1). It has carbon fiber plates sandwiched with a plastic foam core. Its carbon fiber construction ensures that no metal parts are used in the entire treatment area. BrainLAB's ICT is 53 cm wide, 200 cm long, and has a 5 cm thickness of which 2 mm is made up of carbon fiber. It weighs 11.9 kg and can hold a maximum load of 185 kg. There are also a couch extension (headrest) (53cm×23cm×2cm) and an ICT frameless extension (53cm×41.5cm×2cm). The thickness of the carbon fiber in the extension is 2 mm for the top layer and 0.75 mm for the bottom layer. The entire table is designed for remote robotic control capability with 6 degrees of movements including, pitch, roll and yaw.

### B. Novalis ${\text{Tx}}^{\text{TM}}$

Novalis Tx is the product of a joint venture between Varian Medical Systems (Palo Alto, CA, USA) and BrainLAB Inc, capable of providing stereotactic radiosurgery and radiotherapy. It is equipped with a 120 multileaf collimator with 2.5 mm thick central leaves and 5 mm thick outer leaves. Novalis Tx is capable of producing from 6 MV up to 18 MV photons, with multiple electron energies. Its capabilities include BrainLAB ExacTrac with stereo X‐ray^(14)^ and on‐board imager using cone beam CT.

## III. METHODS

Transmission of 6 MV and 18 MV photon beam from a Novalis Tx linear accelerator through the ICT material was measured. A Farmer‐type ionization chamber, Exradin model A12 (Standard Imaging, Middleton, WI, USA) with a collecting volume of 0.65 cc, was used. The chamber was connected to a Max 4000 electrometer (Standard Imaging, Middleton, WI, USA). A delrin $\left(1.42\;g/{\text{cm}}^{3}\right)$ cylindrical buildup cap (1.4 cm thick) was used for the 6 MV, and a brass $\left(8.455\;g/{\text{cm}}^{3}\right)$ cylindrical buildup cap (0.8 cm thick) was used for 18 MV measurement. A brass buildup cap was used because Weber et al.^(15)^ demonstrated that at field sizes less than $20\times 20\;{\text{cm}}^{2}$, a brass buildup cap produces results similar to a tissue equivalent buildup cap such as graphite. The chamber was set up at the isocenter of the linear accelerator (SAD setup of 100 cm).

### A. Attenuation measurement

**Figure 2:**
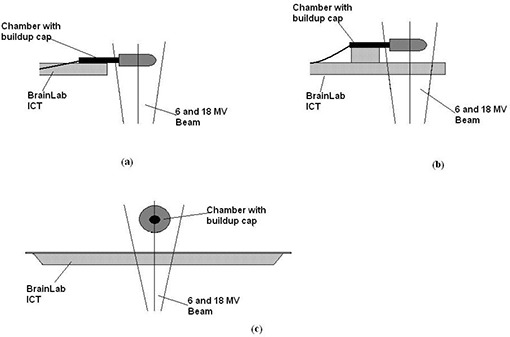
(a) in air ionization measurement using a buildup cap; (b) transmission measurement in the chamber with a buildup cap placed 5 cm above the couch; (c) cross‐sectional view of the measurement through the couch.

To determine the attenuation through the imaging couch top, the couch extension, and ICT frameless extension, the following was done:

- Measurement of ionization in the chamber with an appropriate buildup cap (in air measurement, Fig. 2(a)).
- Another set of measurements with the chamber (with the appropriate buildup cap) placed 5 cm above the couch surface (Figs. 2(b), 2(c)) by securing the chamber holder on a 5 cm thick solid water phantom. This setup was aimed at reducing the forward scatter radiation from the couch. Other studies in attenuation of megavoltage photons have used the 5 cm depth.^(12)^
- Percent transmission was defined as=(Ionization charge through the couch/ionization without the couch)1*00
- Percent attenuation=100−% transmission
- Repetition of the second step, but with the chamber placed 5 cm above ICT frameless extension.
- Repeat measurements for other photon energy.

All measurements were carried out at the same ambient temperature and pressure, so no corrections were required.

### B. The effect of gantry angle on transmission

We also investigated the effect of gantry angles on transmission through the ICT. The angle was initially set at 180° so that the radiation field was normally incident on the couch; hence, the angle of incidence θ, on the couch was 0°. The angle was then rotated counterclockwise in 10° increments towards the plane of the couch. (Note the International Electrotechnical Commission (IEC) convention was used for the angles whereby at zero degree, the gantry is pointing to the floor and, at 180 degrees, it is pointing to the ceiling). No measurements were carried out in the clockwise direction (from 180° to 270°) as the assumption was that any angular dependence would be symmetric, since Spezi and Ferri^(12)^ had previously demonstrated this dependence.

Preliminary measurements had shown an angular dependence of the linear accelerator output, prompting in air measurements at the same gantry angles but this time without the couch. Two field sizes were investigated: $10\times 10\;{\text{cm}}^{2}$ and $5\times 5\;{\text{cm}}^{2}$ ‐ chosen because smaller field sizes (beamlets) are more likely to be used in IMRT than large field sizes. For each setup, at least three repeated measurements were recorded for a dose of 100 monitor units delivered at 600 MU/min, and an average value computed.

## IV. RESULTS

As previously reported,^(13)^ attenuation measurements are considered relative; hence, absolute values are not deemed necessary. In this study, both sets of measurements (with and without couch) experienced the same ambient temperature and pressure. Corrections for temperature and pressure were consequently not applied. ICT frameless extension and couch extension (headrest) were of the same thickness; hence, only measurements for the couch extension were made and these are applicable to the frameless extension.

The output measurements of the Novalis Tx as a function of angle are presented in Table 1. The values are percentage deviation from the output measurement at 0° (gantry facing down). It is evident from Table 1 that the linear accelerator output is dependent on the gantry angle. There was an increase in output with increase in gantry angle for both 6 MV and 18 MV photon energies. The increase in output with increase in gantry angle was also influenced by the field size.

**Table 1:** Angular dependence of the Linear accelerator output (percentage deviation from the gantry angle of zero).

	*6 MV*		*18 MV*	
*Angle*	5×5cm	10×10cm	5×5cm	10×10cm
0	0.00	0.00	0.00	0.00
90	0.49	0.28	0.52	0.52
100	0.55	0.36	0.50	0.64
110	0.67	0.40	0.61	0.71
120	0.76	0.47	0.68	0.79
130	0.78	0.52	0.66	0.82
140	0.85	0.55	0.71	0.76
150	0.88	0.59	0.76	0.82
160	0.89	0.57	0.78	0.83
170	0.92	0.58	0.76	0.83
180	0.88	0.60	0.74	0.78
270	0.32	0.23	0.20	0.27

**Figure 3:**
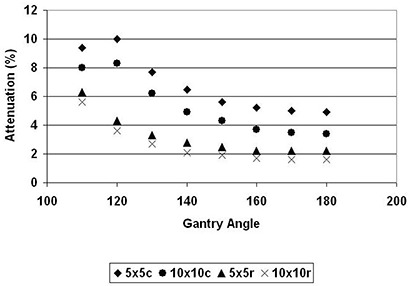
Gantry dependence of the transmission of 6 MV photon beam to the BrainLAB's couch (c) and headrest (r) for $5\times 5\;{\text{cm}}^{2}$ and $10\times 10\;{\text{cm}}^{2}$ field sizes.

The ICT couch and headrest attenuation of 6 MV photon beam for the $5\times 5\;{\text{cm}}^{2}$ and $10\times 10\;{\text{cm}}^{2}$ field sizes are presented in Table 2 and Fig. 3. At normal incidence on the couch, the attenuation was 4.9% and 3.4% for $5\times 5\;{\text{cm}}^{2}$ and $10\times 10\;{\text{cm}}^{2}$ field sizes, respectively. Attenuation varied as a function of the gantry angle. The highest attenuation of 10% was observed at a gantry angle of 120°. As expected, attenuation on the headrest was lower than on the couch. A maximum attenuation for the headrest of 6.3% occurred at gantry angle of 110°. Normal incidence attenuation for the headrest was 2.2% and 1.6% for the $5\times 5\;{\text{cm}}^{2}$ and $10\times 10\;{\text{cm}}^{2}$ field sizes, respectively.

**Table 2:** 6 MV photon beam attenuation by the ICT couch and headrest.

	*Couch*		*Headrest*	
*Angle*	5×5cm	10×10cm	5×5cm	10×10cm
100	0.4	−0.7	0.3	−0.1
110	9.4	8	6.3	5.6
120	10	8.3	4.3	3.6
130	7.7	6.2	3.3	2.7
140	6.5	4.9	2.8	2.1
150	5.6	4.3	2.5	1.9
160	5.2	3.7	2.2	1.7
170	5	3.5	2.2	1.6
180	4.9	3.4	2.2	1.6

**Figure 4:**
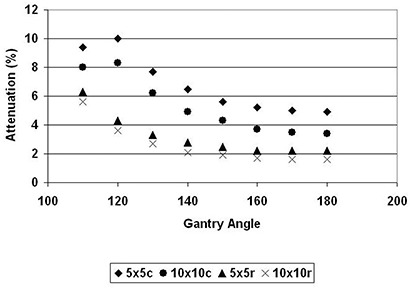
Gantry dependence of the transmission of 18 MV photon beam to the BrainLAB's couch (c) and headrest (r) for $5\times 5\;{\text{cm}}^{2}$ and $10\times 10\;{\text{cm}}^{2}$ field sizes.

The ICT couch and headrest attenuation for 18 MV photon for both the $5\times 5\;{\text{cm}}^{2}$ and $10\times 10\;{\text{cm}}^{2}$ field sizes are presented in Table 3 and Fig. 4. The carbon fiber couch and headrest had negligible attenuation on the 18 MV photon beam when it propagated at normal incidence to the couch. Significant attenuation was, however, observed at oblique incidences. Maximum attenuation for the $5\times 5\;{\text{cm}}^{2}$ field size was 3.7% at 110°. There was no significant field size dependence for 18 MV attenuation.

**Table 3:** 18 MV photon beam attenuation by the ICT couch and headrest.

	*Couch*		*Headrest*	
*Angle*	5×5cm	10×10cm	5×5cm	10×10cm
100	−0.5	−1.3	−0.3	−1.1
110	3.7	3.5	2.3	2.1
120	3.6	3.4	1	0.8
130	2.1	2.1	0.4	0.4
140	1.3	1.4	0.1	0.1
150	0.9	1	0	0
160	0.7	0.9	0	0
170	0.6	0.8	0	0
180	0.6	0.7	−0.1	0

At 100°, the main beam does not propagate through the couch; however, there is a scatter effect from the couch (in‐scattering). This effect is not present without the couch; hence, there is an apparent enhancement at that angle.

## V. DISCUSSION

### A. Linac output variation with angle

There was less than 1% variation in the linear accelerator output as a function of gantry angle. It has been postulated that as the linear accelerator gantry rotates, variation in output intensity is expected due to gantry sag, causing slight movements of the electron optics and head components, and geometric changes in the beam path.^(16)^ It is noteworthy that gantry angle variation of the output was within the tolerance of 1.5% as provided by Varian Medical Systems. As radiation therapy becomes more conformal with the advent of IMRT and IGRT, there will be a need to account for this variation in treatment planning.

### B. ICT couch attenuation

The BrainLAB Imaging couch top is a robust light‐weight and low‐attenuating patient positioning device. It facilitates the implementation of both cone beam CT and orthogonal X‐ray imaging. There is, however, potential for significant beam attenuation through this couch because we observed, for normal incidence, a beam attenuation of 3.4% to 4.9% for 6 MV photon and 0% to 0.7% for 18 MV photons. These values are slightly higher than those reported by other researchers for different carbon fiber tops.^(^ ^11^ ^,^ ^12^ ^,^ ^17^ ^)^ Spezi and Ferri^(12)^ evaluated a Siemens IGRT tabletop and found that for a $10\times 10\;{\text{cm}}^{2}$ field size, a 6 MV photon was attenuated by 2.1%. Gillis et al.^(11)^ evaluated the Sinmed Mastercouch and reported 1.5% attenuation for the $5\times 5\;{\text{cm}}^{2}$ field size for both 6 MV and 18 MV photons. Independently, McCormach et al.^(13)^ also reported a 2.2% 6 MV photon beam attenuation for the Sinmed Posisert couch, for direct incidence, of the $10\times 5\;{\text{cm}}^{2}$ field size.

Our attenuation measurements, however, match those of Munjal et al.^(10)^ and Poppe et al.^(18)^ who report an attenuation coefficient of 0.082 cm^−1^ for 6 MV photon. The BrainLAB ICT has a 4 mm total carbon fiber thickness. If the ICT was just carbon fiber, the expected attenuation, according to the Munjal study, would be 3.23% for 6 MV. We recorded 3.4% to 4.9% for the $5\times 5\;{\text{cm}}^{2}$ and $10\times 10\;{\text{cm}}^{2}$ field sizes, respectively. Any discrepancy between the predicted value and measured value could be attributed to the foam. We measured the CT number using our GE Light speed and found those for carbon fibers to be −540±10 and foam to be −896±6, leading to the conclusion that foam can also contribute to the attenuation of the photon beam (albeit at a lower coefficient).

Attenuation of the photon beam is also influenced by the thickness of the carbon fiber used in the construction of the couch. This is clearly demonstrated by the difference in attenuation between the couch (3.4%−4.9%) and the headrest of the BrainLAB ICT (1.6%−2.2%). The total thickness of the carbon fiber is 4 mm for the couch and 2.7 mm for the headrest and the frameless extensions. The key variation in the commercially available carbon fibers tabletops are the carbon fiber thickness and the material used to sandwich the plies.

### C. Angular dependence of attenuation

Previous studies of photon beam attenuation by carbon fiber couch top were mostly concerned with normal incidence at a gantry angle of 180°. Only limited studies have evaluated the impact of gantry angle on attenuation.^(^ ^10^ ^,^ ^13^ ^,^ ^19^ ^)^ With the advent of IMRT, however, oblique incidences of treatment beams are more commonplace. Traditional conformal treatment of cancers such as esophagus and off‐cord treatment of the lungs also require oblique incidence of the treatment beam. There is consequently a clinical need to evaluate attenuation at such oblique angles. We found that attenuation of the photon beam varies with the gantry angle (Figs. 3 and 4). The attenuation of 6 MV photon by the couch increased from 4.9% to 10% for a $5\times 5\;{\text{cm}}^{2}$ field size, and from 3.4% to 8.3% for a $10\times 10\;{\text{cm}}^{2}$ field size. Attenuation by the headrest also increased from 2.2% at normal incidence to a maximum of 6.3% at 110° gantry angle. Similar increases – from 0.6% to 3.7% – were also observed for 18 MV photon.

**Figure 5:**
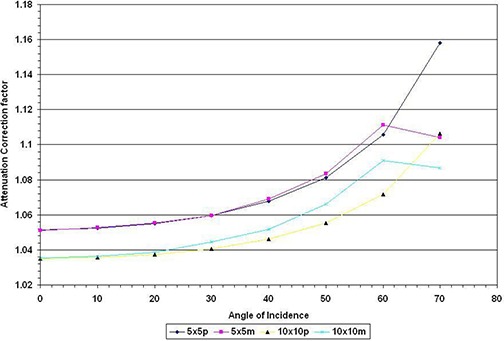
Ratio of measurements without to measurements with couch in the beam expressed as the function of the angle of incidence for $5\times 5\;{\text{cm}}^{2}$ and $10\times 10\;{\text{cm}}^{2}$ field sizes; *p* represents values predicted theoretically and *m* represents measured values.

Apparently, as the angle becomes more oblique, the distance traversed by the beam through the carbon fibers increases. The path length traveled by the photon can be estimated by t/cosθ, where θ is the angle of normal incidence and oblique incidence of the beam (θ=180−gantry angle), and *t* is the thickness of the couch. Using the measured attenuation at normal incidence, one could predict the subsequent attenuation at a different angle 9 (Fig. 5) using Eq. (1):

(1) $$I_{f}=I_{o}e^{\mu t/\cos\theta }$$

We observed a trend similar to McCormark et al.^(13)^ As the angle of incidence increased, the measured attenuation of the radiation beam is greater than predicted by Eq. (1). Our measured values however dropped at angle of incidence of 70° (equivalent to gantry angle of 110°). This is because at that angle, the beam grazed the couch; hence the predicted path length in the couch is actually longer than the path length of the beam in the couch.

McCormack et al.^(13)^ postulate that as the angle of incidence increases, lesser of the forward scatter radiation is recorded at the chamber, giving rise to lower electrometer reading and, therefore, a perceived higher attenuation. Regardless, oblique incidence of radiation will result in higher attenuation than that reported for normal incidence. This will result in more than acceptable reduction in the dose delivered at the tumor target volume.

It has been suggested that a correction be applied to the treatment planning monitor unit (MU) using Fig. 5.^(13)^ Potential caveats however need to be addressed. First, it has been documented that carbon fiber tops lead to loss in skin sparing effect;^(^ ^8^ ^,^ ^20^ ^,^ ^21^ ^)^ hence, any additional compensation for attenuation will therefore lead to further increase in the skin dose. Secondly, it will be difficult to compensate for attenuation when only part of the field intersects the beam. A possible solution would be to incorporate the carbon fiber top into the body contour outline in the treatment planning system. Attenuation could then be calculated along the oblique path and adjusted for it accordingly. This approach has been demonstrated by Myint et al.^(19)^ and Spezi et al.^(22)^ Myint found that incorporating the carbon fiber couch in the patient model reduces the dose error from 10% to less than 2%. Spezi et al. found that IMRT optimization with the Siemens IGRT tabletop results in an increase in MU as much as 37% per beam. However, we could not incorporate CT couch into treatment planning because CT couch is not the same thickness as BrianLAB ICT. Nevertheless, the validity of these solutions is being evaluated at our center.

### D. Energy dependence

We observed that for 18 MV, there was insignificant attenuation by the ICT at normal incidence. Reported attenuation for carbon fibers has been mostly limited to 6 MV. Meara and Langmack^(9)^ studied the attenuation for 5 MV, 6 MV and 8 MV photons and observed a slight increase in transmission (decrease in attenuation) through the carbon fibers with increase in energy. Myint et al.^(19)^ found a significant decrease in attenuation for higher energy. For example, for a $10\times 10\;{\text{cm}}^{2}$ field size, they observed 15.3% attenuation for 6 MV compared to 9.3% for 18 MV, for a beam going through the carbon fiber rails of a MED‐TEC indexed patient positioning system at a gantry angle of 225°.

### E. Field‐size dependence

We found the attenuation through the carbon fibers to be field‐size dependent. 6 MV photon yielded a 4.9% and 3.4% attenuation for $5\times 5\;{\text{cm}}^{2}$ and $10\times 10\;{\text{cm}}^{2}$ field sizes, respectively (see Table 2). Similarly for the headrest, the attenuation for attenuation was 2.2% and 1.6% for $5\times 5\;{\text{cm}}^{2}$ and $10\times 10\;{\text{cm}}^{2}$, respectively. Apparently, higher attenuation is recorded on smaller field sizes and this could be attributed to the effect of scatter contribution. Compared to larger field sizes, smaller field sizes have more out scatter radiation that is not recorded by the chamber.

### F. Clinical implication

It would appear ICT has an impact on the dose delivered to the target when an incident beam traverses the couch. The magnitude of its impact will however depend on the type of treatment, photon energy used, field size, and total number of beams intersecting the couch. There are various clinical situations where the beams will traverse the couch or the headrest. For instance, four field box for the pelvic irradiation, AP/PA for the lungs, head and neck IMRT, prostate IMRT, and brain IMRT. We randomly identified prostate, head and neck, and brain IMRT patients. The dose resulting from optimization by our treatment planning computer (a Pinnacle direct machine parameter optimization, version 8.0M) was used. Dose reduction per gantry angle was approximated on the basis of the same magnitude as the measured attenuation given in Tables 2 and 3. Results of the estimated dose to be delivered to the target, using the ICT or headrest, are presented in Table 4.

**Table 4(a):** Estimate of the impact of using the imaging couch without any corrections for 7 field prostate IMRT treatments. (Computed dose is generated by the treatment planning, and the target dose is that after accounting for attenuation.)

*Angle*	*Computed Dose (cGy*)	*% reduction due to attenuation*	*Target Dose (cGy*)
180	33	4.9	31.4
135	25	7.1	23.2
100	33	0.4	32.9
40	17	0	17
320	17	0	17
260	30	0.4	29.88
225	25	7.1	23.23
Total dose	180		174.6

For prostrate patients, our cancer center uses seven fields at gantry angles listed on Table 4(a). Five of the gantry angles traverse the couch. It is approximated that for 180 cGy prescription, using optimization, 174.6 cGy will be delivered to the target; hence, a total loss of about 3% due to the couch. Similarly for head and neck IMRT, our center uses 9 fields, of which only 4 will traverse the couch. Head, neck and brain use the headrest that has lower attenuation. 177.1 cGy will be delivered to the target for a 180 cGy prescription, incurring only a 1.6% loss (see Table 4(b)). For a brain IMRT, we use 9 fields, of which only 4 will traverse the couch. Using the same computation, for a prescription of 200 cGy, only 197.5 cGy will be delivered to the target, resulting in a dose decrement of 1.3% due to attenuation in the headrest. Evidently, the couch or headrest has a clinical impact on the dose delivered to the target.

**Table 4(b):** Estimate of the impact of using the BrainLAB ICT head extension (headrest) without any corrections for 9 field head and neck IMRT treatment.

*Angle*	*Computed Dose (cGy*)	*% reduction due to attenuation*	*Target Dose (cGy*)
0	16	0	16
40	21	0	21
80	18	0	18
120	25	4.3	23.9
160	23	2.2	22.5
200	20	2.2	19.6
240	21	4.3	20.1
280	19	0	19
320	17	0	17
Total Dose	180		177.1

## VI. CONCLUSIONS

We have demonstrated significant attenuation for the BrainLAB ICT for 6 MV. Attenuation increases as the path length of the beam in the couch increases. Extreme caution has to be exercised, therefore, in the usage of oblique angles for treatment using BrainLAB ICT. There is no significant attenuation for normal incidence for 18 MV. However we observed significant attenuation for oblique angles of incidence. A limitation of this study is that it does not address increased skin dose, which has been reported elsewhere.^(^ ^8^ ^,^ ^20^ ^,^ ^21^ ^)^

## ACKNOWLEDGEMENTS

We would like to thank Valentine Mungyeh for editing the manuscript.
